# Survey on Sildenafil, Tadalafil, and Vardenafil Concentrations in Food Supplements for Erectile Dysfunction

**DOI:** 10.1155/2022/3950190

**Published:** 2022-07-09

**Authors:** Elina Petkova-Gueorguieva, Stanislav Gueorguiev, Hristina Lebanova, Vasil Madzharov, Anna Mihaylova

**Affiliations:** ^1^Department of Health Policy and Management, Faculty of Public Health, Medical Univercity of Sofia, 15 Akademik Ivan Evstratiev Geshov Blvd, 1431 Sofia, Bulgaria; ^2^Department of Pharmaceutical Sciences, Faculty of Pharmacy, Medical University of Plovdiv, 15A Vasil Aprilov Street, 4002 Plovdiv, Bulgaria; ^3^Department of Pharmaceutical Sciences and Social Pharmacy, Faculty of Pharmacy, Medical University—Pleven, 1 Kliment Ohridski Street, 5800 Pleven, Bulgaria; ^4^Medical College, Medical University of Plovdiv, 15A Vasil Aprilov Street, 4002 Plovdiv, Bulgaria

## Abstract

The aim of the study was to analyze the presence of undeclared sildenafil, tadalafil, and vardenafil in food supplements (FSs) for erectile dysfunction. The presence of sildenafil, tadalafil, and vardenafil was determined using the generated ultraviolet (UV)-spectra and mass-spectrometry (MS)-spectra as well as chromatograms produced by the photodiode array (PDA)-detector and ion trap MS-detector. The results were processed by Xcalibur ver. 2.0.7. Fourteen of the 20 analyzed FSs contained undeclared ingredients. Sildenafil was present in 12 of them. Many violations and discrepancies between the label information and the real composition of the FS were identified. 70% of the samples contained undeclared ingredients of an erectile dysfunction medicinal product. The quantities varied within broad limits from 2 mg per tablet to 116.55 mg per tablet. Sildenafil was present in amounts exceeding 16.55 mg that is the maximum recommended dose, thus creating risk of overdose. Besides that, food supplements adulterated with sildenafil analogues are a health risk for consumer's health as there is no evidence of modified sildenafil toxicity. All analyzed FSs were claimed to be 100% natural, not provoking side effects. No information for any FS contained indications of age limits or risk for interaction with other FSs or medical products.

## 1. Introduction

Food supplements (FSs) are concentrated sources of nutrients or other substances with nutritional or physiological effects aiming to contribute to the normal nutritional balance. They are marketed in a preset dose form such as tablets, capsules, dosed liquids, and other drug forms.

The last year evidenced globally and nationally increased interest in food supplements. The global FS market is expected to exceed 220 billion US dollars and to increase by more than 8% by 2022.

Erectile dysfunction (ED, impotence) is a disorder in male sexual function. It is expressed in the inability to achieve or sustain penis erection necessary to realize the sexual act [1, 2]. It not only seriously affects men's and their families' lifestyles but can also be an early sign for a grave disease of the coronary arteries or the peripheral vessels [3]. ED can happen to any man, but it most frequently occurs after the age of 60. Quilter et al. (2017) found that ED risk of men aged 60+ was 3 times higher than that of those aged 40 [4].

The pharmaceutical market offers FS and herbal products designed for men with erectile dysfunction. The compositions of those supplements involve mainly herbs supposed to have a positive effect in the case of ED. Such frequently input herbs are yohimbe, maritime (cluster) pine (*Pinus pinaster*), tongkat ali (longjack) (*Eurycoma longifolia*), Chinese magnolia vine (*Schisandra chinensis)*, goat's head (*Tribulus terrestris)*, barrenwort (*Epimedium*), dehydroepiandrosterone, and L-arginine.

The postproduction quality and quantity control on FS is not mandatory unlike that on drugs. This is the major factor urging many researchers to present alarming reports for undeclared ingredients found in FS [5–8]. The lack of mandatory analytical control and the liberal regulation policy on FS can lead to the input of deliberately undeclared substances, accidental contamination during the production process, and adulterated food supplements.

The most frequently undeclared ingredients in FS for erectile dysfunction are medicinal products such as sildenafil, vardenafil, and tadalafil.

Sildenafil is a powerful selective inhibitor of phosphodiesterase type 5 (PDE5). It is administered for the treatment of erectile dysfunction. Sildenafil is prescribed for the treatment of erectile dysfunction including in men with diabetes and dysfunction caused by medicines intake. It is available in packs of 2 to 4 tablets of 50 mg and 100 mg. The usual starting dose is 50 mg. It is contraindicated for patients who have suffered a brain or heart stroke during the last 6 months, unstable or decompensated heart disease, hypertension with high values of the arterial tension or very low blood pressure, patients with liver or kidney disease, and in case of retinitis pigmentosa [9]. Considering the possible cardiovascular risk, it is recommended to identify the patient's cardiovascular status by a physician-cardiologist [10].

Tadalafil is indicated for the treatment of erectile dysfunction in adult men [11–13]. It is available in packs of 2 to 4 tablets of 10 mg and 20 mg. The usual starting dose is 10 mg. Its mechanism of action is similar to that of sildenafil, and it is a powerful selective inhibitor of PDE5. There are also similar contraindications in intake of tadalafil; for example, there is a possibility of development of a grave life-threatening hypotension in patients who use vasodilators of the nitrate type, and thus it is necessary to allow a 48-hour minimal interval between nitrate and tadalafil intakes [14].

Vardenafil is also administered for the control of erectile dysfunction in elderly men and has the same mechanism of effect [15–17]. It is available in packs of 2 to 4 tablets of 10 mg and 20 mg. The usual starting dose is 10 mg. However, its effect on erectile dysfunction does not depend on patients' age, the severity of concomitant disease, its aetiology, presence of diabetes, or prostatectomy [9]. All three products are contraindicated in the use of nitrate-containing medicines as the combination may cause a potentially dangerous decrease in blood pressure.

The success of selective phosphodiesterase inhibitors (sildenafil, vardenafil, and tadalafil) in the management of erectile dysfunction is used by the manufacturers of herbal food supplements with added medical substances that are not declared on the label and in the leaflet. There exist studies stating that not only these three permitted drugs but also their nonlicensed analogues have been detected in FS. Structurally modified analogues that stay hidden from the patient and the control institutions are yet more frequently input according to certain communications [18]. There is also evidence of imitations of popular trademarks.

On the other hand, erectile dysfunction affects 10% of the men, and very often, because of the discreetness of the issue, men do not ask for medical help but tend to use FS ordered at Internet sites that promise management of the problem and 100% effectiveness of the supplement. Very often those FSs contain the aforementioned drugs (sildenafil, tadalafil, and vardenafil) without stating that on the label or the leaflet and without informing the patient about possible undesired effects and contraindications [19–22]. These circumstances could lead to serious, even fatal consequences due to the intake of such FSs (as it is mentioned in the characteristics of the phosphodiesterase inhibitors).

The Food and Drug Agency (FDA) reported 572 cases in the 2007–2014 period of FSs that contained undeclared active ingredients which could be present as components only in the formulation of a medicinal product [5]. However, 41.6% refer to FSs intended for patients with erectile dysfunction. Over the same period in Europe, 929 analogous cases were reported. In the 2007–2013 period, the Brazilian authorities analyzed 2898 FSs and determined that 180 of them contained undeclared ingredients.

A survey conducted in 2006 in China established the content of the sildenafil analogue in two food supplements for erectile dysfunction [23]. Its structure was similar to that of sildenafil with a single difference in the piperazine part where N-CH_3_ was substituted with N-CH_2_CH_2_OH. This compound is known as hydroxyhomosildenafil. It has not been licensed for use as a drug. Doubtlessly, the intake of those two food supplements is associated with a possible risk for cardiovascular injuries.

By 2012, at least 46 different analogues of sildenafil, tadalafil, and vardenafil detected in FSs for erectile dysfunction had been reported [24]. The number of analogues of sildenafil, tadalafil, and vardenafil identified in FS increased from one in 2003 to 46 in 2011. The authors claimed that the use of food supplements that seemed safe, containing analogues of those drugs, could cause serious undesirable effects because they have not been tested for effectiveness and safety profile. Food supplements adulterated with sildenafil, tadalafil, and vardenafil analogues constitute a risk for consumers' health, but the current state of knowledge about the harm they might have induced is still poor.

According to the information submitted by the American regulatory agency Food and Drug Agency (FDA), since May 2016 four FSs, identified with corresponding names, lot number, and shelf life, contained phosphodiesterase inhibitors, which meant that those supplements have not been licensed as drugs [8]. Sildenafil was not listed on the labels of the products. The FDA warned that the undeclared active ingredient was hazardous for the consumers as they did not suspect sildenafil content in those packages and often chose them in order to avoid the side effects of those drugs for erectile dysfunction. The FDA withdrew those products from the market and informed customers by messages through e-mail as well. The FDA encouraged customers to report online any undesired effects of food supplements via the MedWatch programme for reporting undesired events.

In Bulgaria, only undesired drug reactions are communicated through the online sheet created by the Executive Drug Agency (EDA). The information supplied by the patients is extremely important for detecting unknown undesired reactions to drugs permitted for use in Bulgarian territory [25].

The site of the Bulgarian Food Safety Agency (BFSA) neither enables the registration of information related to FS problems nor incorporates a system for complaints associated with the intake of a certain supplement [26].

In general, the standpoint of the regulatory agencies and experts is that the consumers using the FS are not sufficiently aware of the potential risks at their intake. In the case of an undeclared ingredient in the FS, depending on its nature, serious health effects on the cardiovascular, central nervous, secretory, and reproductive system might occur as well as liver injury of dermatological disorders.

## 2. Experimental

To determine sildenafil, tadalafil, and vardenafil presence/absence, the team purchased 20 FSs randomly from Internet suppliers, social network sources, and pharmacies. The analysis was performed at EDA, Directorate “Drug Analysis.” The tested FSs were blinded, precoded, sealed in individual sterile bags, and submitted to the laboratory with a respective transceiver protocol. The laboratory afterward coded the samples according to their laboratory register. When the samples were delivered back from the laboratory, each one was completed with an individual protocol containing sample description, description of the applied methods; presence/absence of sildenafil, tadalafil, and vardenafil; amount; test conditions, and chromatograms of the samples where contents of an undeclared ingredient were detected.

The presence/absence of sildenafil, tadalafil, and vardenafil was proved by the ultraperformance liquid chromatographic (UPLC) method with the PDA-detector and the mass spectrometric detector (Ph. Eur. 2.2.29 Ph. Eur. 2.2.43). The presence of the three active substances was proved by the generated UV-spectra and MS-spectra as well as by the chromatograms from the PDA-detector and the ion trap MS-detector. The content of each component was calculated based on the generated areas of the test solution, referent solution, and their concentrations. The results from the generated chromatograms were processed by Xcalibur version 2.0.7 software.

## 3. Results and Discussion

The determination of the three vasodilators, selective inhibitors of phosphodiesterase type 5, sildenafil, tadalafil, and vardenafil in the form of a mixture of the referent material was made based on the generated UV-spectra and MS-spectra, as well as chromatograms produced by the PDA-detector and the ion trap MS-detector. The identity criteria for the chromatographic tests according to the EDA in-plant laboratory method (ILM) No. 9 were as follows: retention time (RT) in the sample should constitute 0.95–1.05 of the retention time of the referent material, and the UV-spectra of the sample should coincide with those of the referent material.

The chromatograms and MS-spectra of the 20 FSs analyzed by the team revealed that 14 of the samples contained undeclared ingredients (sample No. 1, 2, 4, 5, 6, 7, 8, 10, 11, 12, 13, 14, 17, and 18). Twelve of them contained the undeclared sildenafil ingredient.

Sample No. 4 did not show a peak in the chromatogram corresponding to sildenafil but matched with its analogue (propoxyphenyl thiosildenafil) (molecular ion with *m*/*z* 390), showing UV-spectra with maximums at *ƛ*1max = 226 nm and *ƛ*2max = 291 nm.

The analysis of sample No. 12 showed the presence of the undeclared sildenafil ingredient. On the chromatograms from the two detector types of sample No. 12, the UV-chromatogram at RT: 12.88 showed a peak that the retention time corresponds to the retention time of the sildenafil standard, generated the UV-spectrum with maximums at *ƛ*1max = 227 nm and *ƛ*2max = 292 nm and the MS^2^-spectrum of sildenafil with a molecular ion at *m*/*z*: 447.16, and the daughter ions of which, formed after decomposition, coincided with those of the referent material.

The analysis of sample No. 2 showed the presence of the undeclared sildenafil ingredient sildenafil. On the chromatograms from the two detector types of sample No. 12, the UV-chromatogram at RT: 12.86 showed a peak that the retention time corresponds to the retention time of the sildenafil standard generated the UV-spectrum with maximums at *ƛ*1max = 227 nm and *ƛ*2max = 292 nm and the MS^2^-spectrum of sildenafil with a molecular ion at *m*/*z*: 432.25, and the daughter ions of which, formed after decomposition, coincided with those of the referent material.

The analysis of sample No. 6 showed the presence of the undeclared sildenafil ingredient. On the chromatograms from the two detector types of sample No. 6, the UV-chromatogram at RT: 12.81 showed a peak that the retention time corresponds to the retention time of the sildenafil standard generated the UV-spectrum with maximums at *ƛ*1max = 227 nm and *ƛ*2max = 292 nm and the MS^2^-spectrum of sildenafil with a molecular ion at *m*/*z*: 446.90, and the daughter ions of which, formed after decomposition, coincided with those of the referent material.

The analysis of sample No. 8 showed the presence of the undeclared sildenafil ingredient. On the chromatograms from the two detector types of sample No. 8, the UV-chromatogram at RT: 12.87 showed a peak that the retention time corresponds to the retention time of the sildenafil standard, generated the UV-spectrum with maximums at *ƛ*1max = 227 nm and *ƛ*2max = 292 nm and the MS^2^-spectrum of sildenafil with a molecular ion at *m*/*z*: 447.32, and the daughter ions of which, formed after decomposition, coincided with those of the referent material.

The analysis of sample No. 14 showed the presence of the undeclared sildenafil ingredient. On the chromatograms from the two detector types of sample No. 14, the UV-chromatogram at RT: 12.91 showed a peak that the retention time corresponds to the retention time of the sildenafil standard, generated the UV-spectrum with maximums at *ƛ*1max = 227 nm and *ƛ*2max = 292 nm and the MS^2^-spectrum of sildenafil with a molecular ion at *m*/*z*: 447.23, and the daughter ions of which, formed after decomposition, coincided with those of the referent material.

The analysis of sample No. 1 showed the presence of the undeclared sildenafil ingredient. On the chromatograms from the two detector types of sample No. 1, the UV-chromatogram at RT: 12.86 showed a peak that the retention time corresponds to the retention time of the sildenafil standard, generated the UV-spectrum with maximums at *ƛ*1max = 227 nm and *ƛ*2max = 292 nm and the MS^2^-spectrum of sildenafil with a molecular ion at *m*/*z*: 447.10, and the daughter ions of which, formed after decomposition, coincided with those of the referent material.

The analysis of sample No. 10 showed the presence of the undeclared sildenafil ingredient. On the chromatograms from the two detector types of sample No. 10, the UV-chromatogram at RT: 12.88 showed a peak that the retention time corresponds to the retention time of the sildenafil standard, generated the UV-spectrum with maximums at *ƛ*1max = 226 nm and *ƛ*2max = 292 nm and the MS^2^-spectrum of sildenafil with a molecular ion at *m*/*z*: 446.90, and the daughter ions of which, formed after decomposition, coincided with those of the referent material.

The analysis of sample No. 11 showed the presence of the undeclared sildenafil ingredient. On the chromatograms from the two detector types of sample No. 11, the UV-chromatogram at RT: 12.89 showed a peak that the retention time corresponds to the retention time of the sildenafil standard, generated the UV-spectrum with maximums at *ƛ*1max = 227 nm and *ƛ*2max = 292 nm and the MS^2^-spectrum of sildenafil with a molecular ion at *m*/*z*: 446.90, and the daughter ions of which, formed after decomposition, coincided with those of the referent material.

The analysis of sample No. 7 showed the presence of the undeclared sildenafil ingredient. On the chromatograms from the two detector types of sample No. 7, the UV-chromatogram at RT: 12.90 showed a peak that the retention time corresponds to the retention time of the sildenafil standard, generated the UV-spectrum with maximums at *ƛ*1max = 226 nm and *ƛ*2max = 285 nm and the MS^2^-spectrum of sildenafil with a molecular ion at *m*/*z*: 446.90, and the daughter ions of which, formed after decomposition, coincided with those of the referent material.

The analysis of sample No. 13 showed the presence of the undeclared sildenafil ingredient. On the chromatograms from the two detector types of sample No. 13, the UV-chromatogram at RT: 12.93 showed a peak that the retention time corresponds to the retention time of the sildenafil standard, generated the UV-spectrum with maximums at *ƛ*1max = 226 nm and *ƛ*2max = 291 nm and the MS^2^-spectrum of sildenafil with a molecular ion at *m*/*z*: 447.18, and the daughter ions of which, formed after decomposition, coincided with those of the referent material.

The analysis of sample No. 17 showed the presence of the undeclared sildenafil ingredient. On the chromatograms from the two detector types of sample No. 17, the UV-chromatogram at RT: 13.17 showed a peak that the retention time corresponds to the retention time of the sildenafil standard, generated the UV-spectrum with maximums at *ƛ*1max = 222 nm and *ƛ*2max = 291 nm and the MS^2^-spectrum of sildenafil with a molecular ion at *m*/*z*: 453.65, and the daughter ions of which, formed after decomposition, coincided with those of the referent material.

The analysis of sample No. 18 showed the presence of the undeclared sildenafil ingredient. On the chromatograms from the two detector types of sample No. 18, the UV-chromatogram at RT: 13.17 showed a peak that the retention time corresponds to the retention time of the sildenafil standard, generated the UV-spectrum with maximums at *ƛ*1max = 226 nm and *ƛ*2max = 289 nm and the MS^2^-spectrum of sildenafil with a molecular ion at *m*/*z*: 457.31, and the daughter ions of which, formed after decomposition, coincided with those of the referent material.

The analysis of sample No. 5 showed the presence of an undeclared ingredient, tadalafil. The chromatograms of sample No. 5 at RT: 18.84 generated UV-spectra with maximums at *ƛ*1max = 222 nm and *ƛ*2max = 291 nm and the tadalafil MS-spectrum: 302.03, 268.14, 262.06, 250.06, and 134.95.

The generalized results of the survey on the presence/absence of sildenafil, tadalafil, and vardenafil and the amount of the detected undeclared ingredient in the 20 tested samples are presented in Table 1. The analytical tests were conducted observing the strict requirements of good laboratory practice under the conditions of a laboratory with a license for similar tests.

It should be noted that many violations and discrepancies of the information on the label of the FS and its composition have been detected. Thus, the package of the food supplement of sample No. 4 claimed that the product was 100% natural based only on herbs and contained 67% ginseng, Gingko biloba, and Epimedium, but the chromatographic analysis showed also the presence of an unknown undeclared chemical substance, identified chemically as propoxyphenyl thiosildenafilin in the amount of 25.5 mg per tablet.

The package of FS No. 12 had no label in Bulgarian; the only inscriptions were the name of the product, amount 120 mg and an inscription in English stating that the product was natural. The manufacturer indicated that this FS contained 120 mg yohimbine. It is known that in combination with antistenocardin nitrates this FS containing yohimbine can cause fatal events for the consumer. For the same sample, the analytical tests determined the presence of an undeclared sildenafil ingredient in the amount of 116.55 mg/tabl. It is well known that the maximal dose of the medicinal product sildenafil is set to 100 mg.

Sildenafil was detected in sample No. 2, 6, 8, and 14 in doses 91.5 mg, 93.3 mg, 96.5 mg, and 94.4 mg per tablet, respectively, and this fact was not stated on the package. All four products are marketed through Internet shops with numerous positive comments stimulating their sale. The undeclared sildenafil ingredient in the information sheet creates risk from this FS, which is characteristic of the drug itself. The contraindications and undesired effects of sildenafil are well known and that means that the FS might turn out to be inappropriate for the particular consumer and even pose a threat to their life and health. All four products do not have the attached leaflet, label in Bulgarian, and dosage on the package.

The analytical data for FS sample. No. 1, 10, and 11 ordered from an American Internet site showed an undeclared sildenafil ingredient in amounts, respectively, 79.9 mg, 71.5 mg, and 74.8 mg per tablet. The names of the three food supplements are not listed in the online FDA sheet for blocked and withdrawn from the market food supplements. The packages of the three products showed a similar composition: Epimedium 90%, ginseng, Gingko biloba, Schisandra, maca, calcium, zinc, and L-arginine. For sample No. 1, it was noted that the product was 100% natural, and for sample No. 10, it was noted that it had no side effects and eliminated impotence.

Sample No. 7, 13, 17, and 18 also contained the undeclared sildenafil ingredient in concentrations of 12.9 mg/tabl., 12.94 mg/tabl., 2.2 mg/caps., and 3.4 mg/caps., respectively. The label of the FS of samples 17 and 18 warned that they were a powerful aphrodisiac. The composition of those FSs according to the label on the package was as follows: St. John's wort: stalk, ginseng root, goat's head: stalk, verbena: stalk, cotton thistle bloom, valerian: root, ginger: roots, Gingko biloba: stalk, zinc, and pollen. The two supplements had a leaflet and label in Bulgarian and were sold in the national pharmacy network.

The results for sample No. 5 showed tadalafil in a dose of 19.5 mg/capsule, which was undeclared on the manufacturer's label. The maximal admissible dose of tadalafil is 20 mg. This meant that the food supplement contained the same amount of the active substance as the drug and that was not permitted. The inscription on the product package provided a false statement that the supplement contained yohimbine.

## 4. Conclusions

The performed analytical tests for determination of drug substances used for the treatment of erectile dysfunction (sildenafil, tadalafil, and vardenafil) showed the following:

The majority of the tested 30 FS samples (70%) contained undeclared ingredients of a medical product administered for the management of erectile dysfunction. These were sildenafil (40% of the cases) and tadalafil (one sample No. 5) and another sample (No. 4) that contained the chemical analogue of sildenafil propoxyphenyl thiosildenafil (25.5 mg/tabl.). The amount of sildenafil varied in a broad range from 2 mg/tabl. to 116.55 mg/tabl. (sample No. 12). This undeclared ingredient was in an amount exceeding 16.55 mg that is the maximal admissible dose for the medicine, creating a hazard of overdose. Besides that, food supplements adulterated with sildenafil analogues present a risk for customers' health because the toxicity of modified sildenafil is not known.

For all tested food supplements, it was noted that the products were 100% natural and did not provoke side effects. No age restrictions or risk from interactions with other FS or food products were provided for any of the tested supplements. The majority of FSs (18 of 20 tested) did not have the enclosed label in Bulgarian that contradicted the national regulations managing their use in the Republic of Bulgaria.

## Figures and Tables

**Table 1:** Generalized results for determination of the presence/absence of sildenafil, tadalafil, vardenafil, unknown ingredients, and their respective amounts.

Sample No.	Lab sample	Sildenafil	Vardenafil	Tadalafil	Unknown
1	2688	79.9 mg per tabl.	—	—	—
2	2689	91.5 mg per tabl.	—	—	—
3	2690	—	—	—	—
4	2691	—	—	—	25.5 mg propoxyphenyl thiosildenafil
5	2692	—	—	19.5 mg per tabl.	—
6	2693	93.3 mg per tabl.	—	—	—
7	2694	15.7 mg per tabl.	—	—	—
8	2695	96.5 mg per tabl.	—	—	—
9	2696	—	—	—	—
10	2697	71.5 mg per tabl.	—	—	—
11	2698	74.8 mg per tabl.	—	—	—
12	2699	116.55 mg per tabl.	—	—	—
13	2700	17.4 mg per tabl.	—	—	—
14	2701	94.4 mg per tabl.	—	—	—
15	2702	—	—	—	—
16	2703	—	—	—	—
17	2704	2.2 mg per tabl.	—	—	—
18	2705	3.4 mg per tabl.	—	—	—
19	2706	—	—	—	—
20	2707	—	—	—	—

## Data Availability

Data are contained within the article.

## Supplementary Materials

Supplementary Materials The supplementary file contains the graphical table of the content of the manuscript.
