# Supplementary material for: Survey on Sildenafil, Tadalafil, and Vardenafil Concentrations in Food Supplements for Erectile Dysfunction
Source: Int J Anal Chem. 2022 Jul 9;2022:3950190. doi: 10.1155/2022/3950190 (PMC9288292; doi:10.1155/2022/3950190)
Supplement: Supplementary Materials — The supplementary file contains the graphical table of the content of the manuscript. [file 3950190.f1.pdf]

The performed analytical tests for determination of drug substances showed 70% of the tested 20 Food Supplements samples contained undeclared ingredients of a medical product administered for the management of erectile dysfunction. Food supplements adulterated with sildenafil analogues present a risk for customers' health because the toxicity of modified sildenafil is not known.

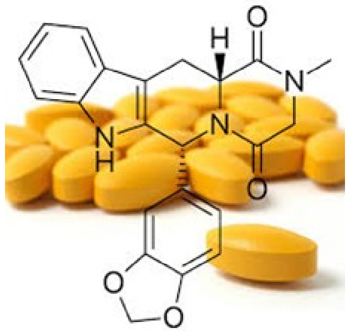

Survey on Sildenafil, Tadalafil, and Vardenafil concentrations in food supplements for erectile dysfunction

Elina Petkova-Gueorguieva,

Stanislav Gueorguiev, Hristina Lebanova,

Vasil Madzharov, Anna Mihaylova*
